# Supplementary material for: The interactome of intact mitochondria by cross-linking mass spectrometry provides evidence for coexisting respiratory supercomplexes
Source: Mol Cell Proteomics. 2017 Dec 8;17(2):216–32. doi: 10.1074/mcp.RA117.000470 (PMC5795388; doi:10.1074/mcp.RA117.000470)
Supplement: Supplemental Data [file supp_RA117.000470_133922_0_supp_23978_fzffwf.zip › spectra_annotation/mito_spectra_annotation/406-1-3-1-9-1.pdf]

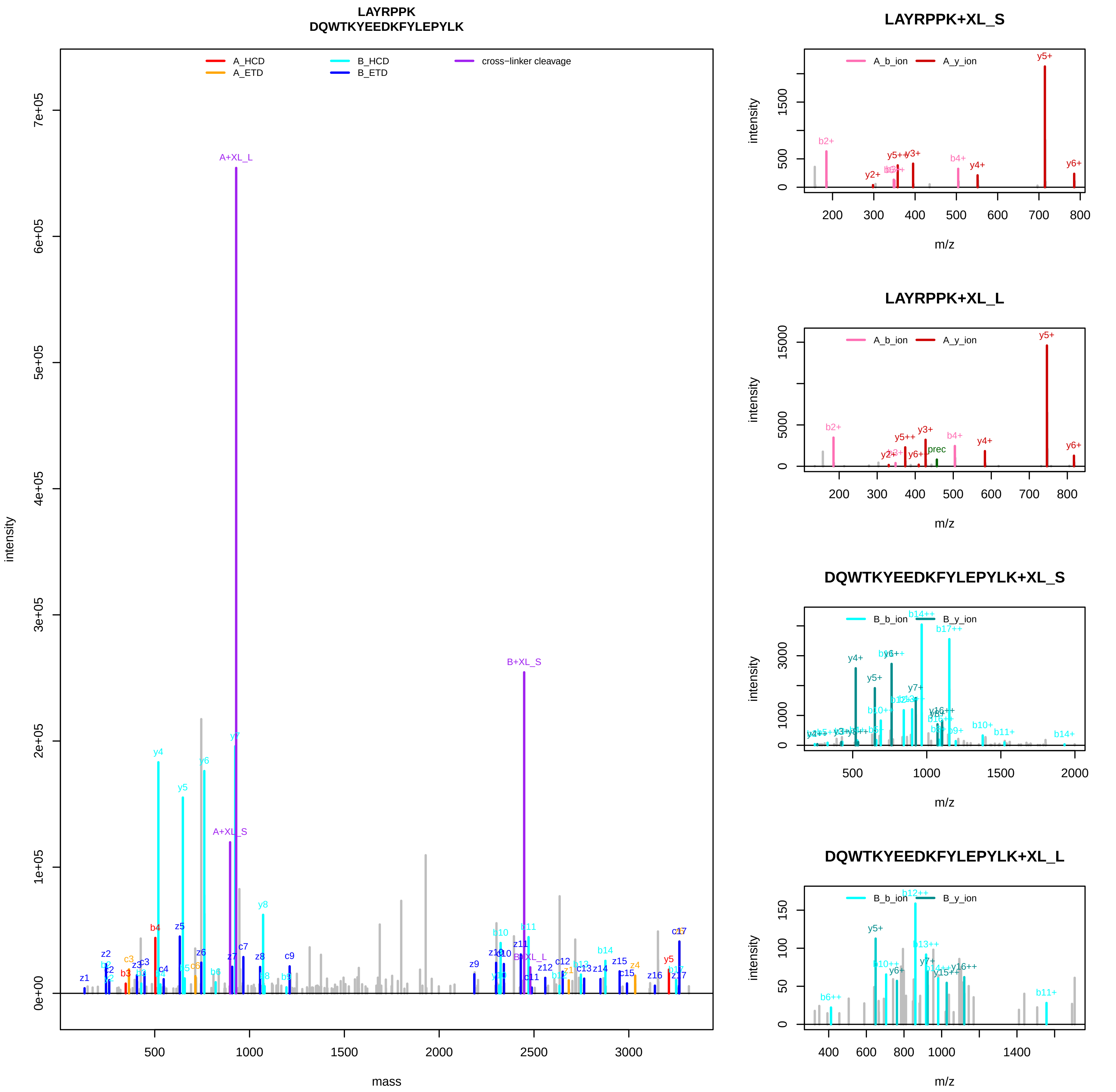

LAYRPPK
DQWTKYEEDKFYLEPYLK
A_HCD
A_ETD
B_HCD
B_ETD
cross−linker cleavage
A+XL_L
B+XL_S
A+XL_S
B+XL_L
intensity
mass
0e+00
1e+05
2e+05
3e+05
4e+05
5e+05
6e+05
7e+05
500
1000
1500
2000
2500
3000
y4
y5
y6
y7
y8
b4
z5
z6
z7
z8
z9
c7
c9
b10
b11
z11
c11
z12
c12
c13
z14
b14
z15
c15
z16
y5
c17
z17
LAYRPPK+XL_S
LAYRPPK+XL_L
DQWTKYEEDKFYLEPYLK+XL_S
DQWTKYEEDKFYLEPYLK+XL_L
A_b_ion
A_y_ion
B_b_ion
B_y_ion
m/z
b2+
b4+
y2+
y3+
y4+
y5+
y5++
y6+
prec
y4+
y5+
y6+
y7+
y16++
b10+
b11+
b14+
b14++
b17++
b13++
b12++
b11+
b6++
y15++
